# A Quantitative Assessment of Costimulation and Phosphatase Activity on Microclusters in Early T Cell Signaling

**DOI:** 10.1371/journal.pone.0079277

**Published:** 2013-10-30

**Authors:** J. Joris Witsenburg, Heike Glauner, Jörg P. Müller, Johannes M. M. Groenewoud, Günter Roth, Frank-Dietmar Böhmer, Merel J. W. Adjobo-Hermans, Roland Brock

**Affiliations:** 1 Department of Biochemistry, Nijmegen Centre for Molecular Life Sciences, Radboud University Nijmegen Medical Centre, Nijmegen, The Netherlands; 2 Institute for Molecular Cell Biology, Jena University Hospital, Jena, Germany; 3 Department of Medical Technology Assessment, Radboud University Nijmegen Medical Centre, Nijmegen, The Netherlands; 4 Laboratory for MEMS Applications, Department of Microsystems Engineering (IMTEK), Albert Ludwigs University, Freiburg, Germany; 5 BIOSS Centre for Biological Signalling Studies, Albert Ludwigs University, Freiburg, Germany; Tulane University, United States of America

## Abstract

T cell signaling is triggered through stimulation of the T cell receptor and costimulatory receptors. Receptor activation leads to the formation of membrane-proximal protein microclusters. These clusters undergo tyrosine phosphorylation and organize multiprotein complexes thereby acting as molecular signaling platforms. Little is known about how the quantity and phosphorylation levels of microclusters are affected by costimulatory signals and the activity of specific signaling proteins. We combined micrometer-sized, microcontact printed, striped patterns of different stimuli and simultaneous analysis of different cell strains with image processing protocols to address this problem. First, we validated the stimulation protocol by showing that high expression levels CD28 result in increased cell spreading. Subsequently, we addressed the role of costimulation and a specific phosphotyrosine phosphatase in cluster formation by including a SHP2 knock-down strain in our system. Distinguishing cell strains using carboxyfluorescein succinimidyl ester enabled a comparison within single samples. SHP2 exerted its effect by lowering phosphorylation levels of individual clusters while CD28 costimulation mainly increased the number of signaling clusters and cell spreading. These effects were observed for general tyrosine phosphorylation of clusters and for phosphorylated PLCγ1. Our analysis enables a clear distinction between factors determining the number of microclusters and those that act on these signaling platforms.

## Introduction

The formation of membrane-proximal protein clusters upon engagement of the T cell receptor (TCR) is a hallmark of early T cell signaling [1], [2], [3]. Cluster formation is the result of protein interactions, driven by phosphorylation of immunoreceptor tyrosine-based activation motifs (ITAMs) in the TCR complex itself and of tyrosines in scaffolding proteins such as the linker for activation of T cells (LAT) [4], [5], [6], [7] and reorganization of the cytoskeleton [8] but the exact mechanisms remain to be further elucidated [9]. These protein clusters represent the molecular platforms of early T cell signaling and ultimately coalesce to form an immunological synapse (IS) [2], [10], [11], [12], [13], [14], [15], [16], [17].

Besides the TCR, costimulatory receptors are of vital importance for T lymphocyte functioning. Cluster of differentiation 28 (CD28) provides the most prominent costimulatory signal and regulates cytokine production, inhibits apoptosis and is required for full T cell activation [18], [19], [20]. CD28 signaling occurs primarily via Phosphatidylinositol 3-kinase (PI3K)-dependent pathways [21], [22], [23], [24], [25], [26], [27]. One of the downstream effectors is phospholipase C-γ1 (PLCγ1) for which CD28 costimulation leads to increased activation and tyrosine phosphorylation [28], [29].

Numerous studies have addressed the role of CD28 in T cell signaling and activation. Manz et al. [30] have even shown that CD28 costimulation decreases the number of engaged peptide-major histocompatibility complexes (pMHCs) per TCR cluster required for T cell activation. Additionally, CD28 has recently been observed to form microclusters that colocalize with TCR clusters upon stimulation with CD80. CD28 subsequently recruits protein kinase C θ (PKCθ) clusters and both CD28 and PKCθ clusters migrate to subregions of the central supramolecular activation cluster (cSMAC) that are distinct from TCR subregions [31]. Importantly, however, the quantitative impact of CD28 costimulation on cluster phosphorylation has not been addressed so far.

Procedures for T cell stimulation incorporating receptor ligands on planar surfaces have proven to be highly powerful in analyzing the dynamics and molecular composition of protein microclusters in a highly defined manner [11]. The incorporation of TCR ligands into lipid bilayers has been key to developing the molecular concept of IS formation [2], and has among others been applied to analyzing the delivery of cytolytic granules and the formation of SRC family kinase microclusters upon TCR engagement on cytotoxic T lymphocytes [32] and signaling induced by viral envelope proteins [33]. The latter study also highlights the advantages that planar-supported substrates offer for quantitative analyses of signaling.

Conversely, microstructured surfaces have been employed to elucidate the molecular mechanisms that underlie the formation of the specific geometric arrangement of the IS [34] as well as the role of specific patterns in the arrangement of stimuli and costimuli in generating a T cell response [35]. The latter study employed microcontact printing for the generation of various patterns of TCR and CD28 stimuli.

Microcontact printing is a robust method for the generation of microstructures of functional proteins in various geometries in micrometer dimensions [36], [37]. Through printing of stripe patterns, functional analysis of different stimuli has been conducted side-by-side for single cells [38].This side-by-side arrangement of stimuli is of particular interest for quantitatively addressing the impact of costimulation on protein cluster formation and tyrosine phosphorylation. Here we describe an accessible procedure that combines microcontact printing, confocal microscopy, high-content image analysis and statistics to study, in parallel, the effect of different stimuli on tyrosine phosphorylation, cluster formation and membrane spreading during early T cell signaling. Within this setup we additionally include the simultaneous analysis of two different cell types and cells with different levels of receptor expression. We demonstrate that the main effect of CD28 costimulation is an increase in the number of microclusters formed as well as the formation of a larger contact area with the stimulating surface.

Moreover, we address the impact of deficiency of SH2-containing protein tyrosine phosphatase 2 (SHP2) on cluster formation. SHP2 is a cytoplasmic protein-tyrosine phosphatase (PTP) that is ubiquitously expressed [39]. Intriguingly, unlike its close relative SHP1, which is widely accepted as a negative regulator of T cell signaling [40], SHP2 has been implicated in both, the inhibition of T cell signaling [41], [42], [43], [44], as well as sustained activation of the mitogen-activated protein kinase (MAPK) pathway by the TCR [40], [45] and many growth factor and cytokine receptors [46]. The T cell signaling proteins PLCγ and PI3K might be directly regulated by SHP2 since it has been shown that these proteins and SHP2 bind to growth factor receptor-bound protein 2 (GRB2)-associated binding protein (GAB)-family adapter proteins which are activated upon activation of T and B cell receptors as well as insulin, growth factor and cytokine stimulation [47], [48], [49]. When addressing the impact of SHP2 on the phosphorylation of signaling microclusters, we show that the deficiency of this PTP leads to a significant increase in overall phosphotyrosine levels and, more specifically, phosphorylation of PLCγ.

## Materials and Methods

### Reagents

Reagents were purchased from Carl Roth (Karlsruhe, Germany) unless otherwise specified. αCD3 (mouse monoclonal IgG2a, clone OKT3) and αCD28 (mouse monoclonal IgG2a, clone 9.3) antibodies were kindly provided by Prof. Dr. Gundram Jung (Department of Immunology, University of Tübingen, Germany). The unspecific mouse IgG2a isotype antibody (clone UPC 10) was purchased from Sigma-Aldrich (Deisenhofen, Germany), the αphosphotyrosine antibody (mouse monoclonal IgG1, clone P-Tyr-100) from Cell Signaling Technology (Leiden, The Netherlands) and αpY783-PLCγ1 (rabbit polyclonal, sc-12943-R) from Santa Cruz Biotechnology (Heidelberg, Germany). The Celltrace CFSE cell proliferation kit containing the carboxyfluorescein diacetate succinimidyl ester (CFDA-SE), Zenon mouse IgG labeling kits and secondary Alexa Fluor-conjugated antibodies were obtained from Molecular Probes, Invitrogen (Breda, The Netherlands). The αIL2 antibodies (cat. 555051 and cat. 555040) and streptavidin-HRP (cat. 554066) were purchased from BD Pharmingen (Erembodegem, Belgium) and the TMB substrate solution from Thermo Scientific (Etten-Leur, The Netherlands).

### Cell Culture

The Jurkat T cell leukemia line (ACC-282) was acquired from the DSMZ (Braunschweig, Germany). Additionally, Jurkat E6.1 SHP2 knock-down cells (SHP2 KD) (see below) were compared to unmodified Jurkat E6.1 T cells (TIB-152, ATCC) termed ‘wild type’ (wt) in this work.

Cells were cultured in RPMI 1640 with stable glutamine and 2.0 g/l NaHCO_3_ supplemented with 10% heat-inactivated fetal bovine serum (FBS) at 37°C and 5% CO_2_ under humidified conditions (medium and serum were both from PAN biotech GmbH, Aidenbach, Germany). Cultures were passed every 2–3 days and grown to densities of on average 7 • 10^5^ cells/ml.

### Cell Transfection

5 • 10^6^ Jurkat cells (ACC-282) in 100 µl serum free RPMI medium were transfected with 5 µg CD28-GFP (RG211318; OriGene Technologies Rockville, MD, USA) in a 2 mm electroporation cuvette (Cell Projects Limited, Kent, UK). Transfection was performed by electroporating the cells at 0.18 kV, 960 µF and 200 Ω (Gene Pulser; Bio-Rad Laboratories, Veenendaal, The Netherlands). The cells were then transferred to 5 ml RPMI medium with 5% FBS and incubated at 37°C for 48 h. After the first 24 h an additional 5 ml of medium with 5% FBS was added to the cells.

### Jurkat E6.1 SHP2 Knock-Down Cells

Plasmid pLKO.1 vectors encoding five nonvalidated shRNA targeting sequences for *ptpn11* (SHP2) were obtained from Sigma-Aldrich (Mission shRNA lentivirus mediated transduction system, SHGLY-NM_002834.3). Targets were validated using transduction of lentiviral particles into 293T cells (ACC 635, DSMZ). With shRNA NM_002834.3-1570s1c1 (targeting sequence CGCTAAGAGAACTTAAACTTTC) a down regulation of SHP2 level to 10% was obtained on western blot (data not shown). For production of lentiviral particles 293T cells were transiently transfected with the pLKO.1-derivative plasmid carrying shRNA NM_002834.3-1570s1c1 in combination with pRev, pEnv-VSV-G and pMDLg using polyethyleneimine (PEI; described recently by Arora et al. [50]). Jurkat E6.1 cells were infected three times with the pseudotyped particles in the presence of 8 µg/ml polybrene (1,5-dimethyl-1,5-diazaundecamethylene polymethobromide, Sigma-Aldrich) for 8, 16, and 24 h. Selection of cells with 2 µg/ml puromycin was started 48 h after transduction.

### Microcontact Printing

Microstructured master templates for the fabrication of poly(dimethylsiloxane) (PDMS) stamps were produced using photolithography [36]. The microstructures were designed in autoCAD 2007 (Autodesk, München, Germany) and ordered as laser-written chromium masks (ML&C, Jena, Germany). A silicon wafer coated with a 2.5 µm thick ma-P 1225 photoresist (Microcoat, Berlin, Germany) was microstructured via photolithography with the chromium masks in a cleanroom facility. After resist development and a hardbake (95°C over 1 h) the microstructured master was finalized with a protective silane coating of low-pressure vapor-deposited (3,3,3-Trifluoropropyl)-trichlorosilane (ABCR, Karlsruhe, Germany).

Stamps were generated by mixing an elastomer base and a cross-linking agent (Sylgard 184 silicone elastomer kit, Dow Corning, Wiesbaden, Germany) in a 10∶1 ratio (w/w). The degassed prepolymer was poured onto the silicon master and cured at 65°C overnight. The PDMS layer was demoulded and cut into individual 8×8 mm stamps. Stamps were coated for 1 h at RT with 100 µl of in total 107 µg/ml antibody solution. These solutions comprised of 7 µg/ml goat αguinea pig Alexa Fluor 647 for visualization of stamped features, 75 µg/ml unspecific IgG2a for titration of the stimulus and lastly a stimulus of either 25 µg/ml αCD3, 25 µg/ml αCD28 or a combination of 12.5 µg/ml αCD3 and 12.5 µg/ml αCD28. Additionally, control stripes were stamped using antibody solutions in which the stimulus was replaced by an additional 25 µg/ml unspecific IgG2a. Meanwhile, microscope slides (75×25×1 mm) were cleaned through rubbing with demineralized water, rinsing with 70% ethanol and acetone and finally dried in a stream of filtered nitrogen. Coated stamps were rinsed with demineralized water, dried with filtered nitrogen and brought into contact with microscope slides for a few seconds. After careful removal of the stamp from the slide an adhesive frame of 1 × 1 cm (In situ frame, Peqlab; Erlangen, Germany) was stuck around the stamped area as an incubation chamber. Parts of the surface that had not been in contact with stamp features were functionalized through a 30 min incubation with 100 µl of a 20 µg/ml antibody solution comprised of 15 µg/ml unspecific IgG2a and a stimulus of either 5 µg/ml αCD3, 5 µg/ml αCD28 or a combination of 2.5 µg/ml αCD3 and 2.5 µg/ml αCD28. Control surfaces were coated with 20 µg/ml unspecific IgG2a only. After a wash step with 150 µl PBS, slides were blocked with 1% BSA in PBS for 30 minutes. Before cell seeding slides were washed with 150 µl PBS once more (Reviewed in [37]).

### Cell Stimulation and Immunocytochemistry

After either transfection or (mock) labeling of cells with 1 µM CFDA-SE at 1•10^6^ cells/ml according to the supplier’s protocol, cells were serum starved in order to reduce background levels of phosphorylation. 1•10^5^ serum starved cells in RPMI 1640 medium were seeded onto functionalized glass surfaces and stimulated for 10 min at 37°C before they were fixed with 3% (w/v) paraformaldehyde (Merck, Darmstadt, Germany) for 10 min at 4°C followed by 15 min at RT, washed three times with PBS for five min and permeabilized with saponin buffer (0.1% saponin, 0.1% BSA in PBS) for 15 min. The αphosphoTyr antibodies were fluorophore conjugated by formation of non-covalent immunocomplexes with Zenon Alexa Fluor 546 labeling kits following the supplier’s protocol, diluted in saponin buffer and incubated with the cells for 1 h. Alternatively the cells were incubated with 2 µg/ml αpY783-PLCγ1 in saponin buffer for 1 h, washed with saponin buffer for 5 min three times and incubated with 4 µg/ml goat αrabbit Alexa Fluor 546 in saponin buffer for 1 h. Finally, samples were washed three times (PBS, 5 min) and coverslips were mounted using Mowiol mounting medium (Merck; [51]).

### Microscopy and Image Analysis

Images were acquired with a TCS SP5 confocal laser scanning microscope equipped with an HCX PL APO 63× 1.2 N.A. water immersion lens and using the 488 nm line of an argon-ion laser, a 561 nm HeNe and a 633 HeNe laser (Leica, Rijswijk, The Netherlands) according to the used fluorophores and lateral sampling rates of 120 nm. All images had a size of 2048 × 2048 pixels.

The fluorescence intensity of cellular areas at the contact plane of cells and functionalized glass was analyzed in individual confocal slices acquired with a pinhole diameter of 1 Airy unit, using ImageJ [52] with self-written macros (Macro S1 & Macro S2). Binary masks of the stripe image and the image of immunolabeled cells were generated. These masks and inverted duplicates thereof were combined and used to measure the integrated intensities of the immunolabel in cells on different surfaces. The masks were further used to determine the size of the surface areas and, in combination with the integrated intensities, provided the mean intensity of cellular pixels. The values of surfaces lacking cells were used for background correction during data processing.

For the discrimination between different cell types, binary masks were generated for CD28-GFP-high or CFSE-labeled cells and, combined with the immunofluorescence masks representing all cells, for the CD28-GFP-low or unlabeled cell population. Cluster-selective masks were generated from the image of the phosphotyrosine signal using the local maxima function of ImageJ and self-written code. The quantities of labeled and unlabeled cells were determined manually using the cell masks and transmission images.

### Data Transformation and Statistical Tests

In the microcontact printing experiments, for each image, the data was normalized by dividing the value of a specific cell type on a specific surface by the average value of all combinations of cell types and surfaces. This enabled pooling of the data sets of different experiments for statistical analyses. Based on histograms of the data and one-sample Kolmogorov–Smirnov tests it was confirmed that data sets of all subgroups could be considered to be normally distributed. Additionally, Levene’s test for equality of variances justified the comparison of populations using two-sample T tests when only the effect of cell type had to be determined and two-way factorial ANOVAs when both the effect of stimulus and cell type had to be measured. To determine whether cell populations had spreading preferences for a certain stimulus, surface-preference-scores (see Results) were subjected to one-sample T tests with a test value of 1.

Generally, null hypotheses were rejected when p-values were below 0.05 but Bonferroni corrections were applied where necessary. Statistical tests were performed using SPSS 16.0 for Windows (SPSS Inc., Chicago, IL, USA) and IBM SPSS Statistics, version 20 (IBM Corp., Armonk, NY, USA).

### Flow Cytometry

Unspecific IgG2a, αCD3 or αCD28 antibodies were conjugated with either Zenon Alexa 488 or Zenon Alexa 647 according to the supplier’s instructions. 1•10^5^ cells were incubated with the fluorescently labeled antibodies for 1 h at room temperature and washed three times in PBS. To prevent exchange of the non-covalently bound Zenon reagent between the primary IgG2a antibodies, the cells were fixed with 3% paraformaldehyde for 10 min at room temperature and washed in PBS before analysis using a FACSCalibur flow cytometer (Becton Dickinson, Beda, The Netherlands) counting at least 2.5•10^4^ events per sample.

### IL2 ELISA

Wells of a Microlon 96-well flat bottom plate (Greiner Bio-One, Alphen aan den Rijn, The Netherlands) were coated overnight at 4°C with 1 µg/ml αCD3, 1 µg/ml αCD28 or a mixture of 1 µg/ml αCD3 and 1 µg/ml αCD28 in PBS (100 µl per well). Additional wells were treated with PBS only, either for negative controls or stimulation of cells with phorbol myristate acetate (PMA) and ionomycin. The plate was blocked for 30 min with 4% BSA in PBS and washed with RPMI medium. 1•10^5^ cells were seeded per well and stimulated for 22 h at 37°C, 5% CO_2_ and under humidified conditions. Positive control samples were incubated with 12.5 ng/ml PMA and 500 ng/ml ionomycin. IL2 expression was determined through a sandwich ELISA using the reagents mentioned above. Plates were measured on a Benchmark Plus microplate spectrophotometer (Bio-Rad Laboratories). ELISA results were analyzed with two-way factorial ANOVAs and Bonferroni post-hoc tests.

## Results

### Cells with high levels of CD28 expression have increased surface contact areas but lower local tyrosine phosphorylation when stimulated with αCD28 on microstructured surfaces

**Figure 1:**
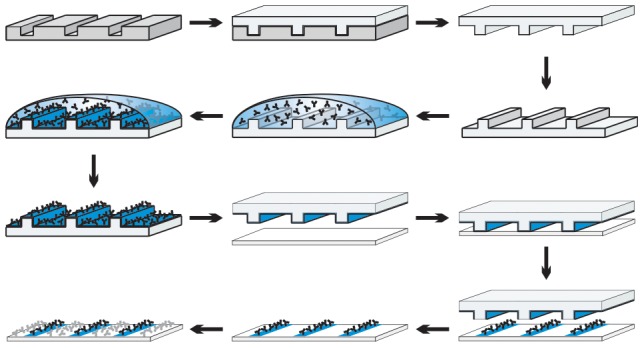
Protocol for microcontact printing. A microstructured silicon master is used as a template for the generation of PDMS stamps. The stamp is coated with antibodies, including a fluorescently labeled indifferent antibody for visualization of stamped features. Stamping transfers a monolayer of antibodies to a clean microscope slide. The areas in between stamped patterns are coated by incubation (‘overlay’) with a second antibody solution. Finally, the surface is blocked with BSA.

We first aimed to determine to what extent different expression levels of the CD28 coreceptor result in different levels of T cell activation. On one hand these experiments served the validation of microcontact printing for quantitative analyses, on the other we intended to compare TCR receptor engagement and the CD28 costimulus in the induction and distribution of tyrosine phosphorylation. One stimulus was transferred onto cleaned glass surfaces by stamping, the other stimulus by incubation with a solution containing the stimulating antibody (termed ‘overlay’ in this work; Fig. 1). It has been shown previously that in this manner each part of the surface contains only one type of stimulus [38].

**Figure 2:**
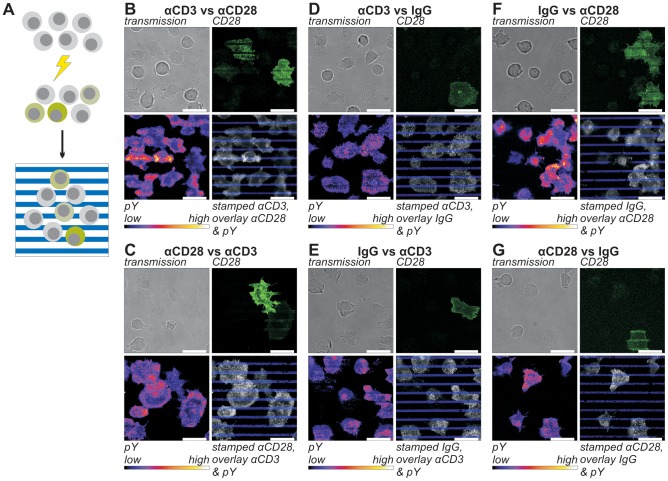
The effect of CD28 expression and segregated, stripe-shaped stimuli on tyrosine phosphorylation. The effect of receptor expression on signaling was studied using CD28-GFP transfected Jurkat ACC-282 T cells. After electroporation, cells were cultured for 48 h, serum starved for 6 h and then incubated on striped stimulatory surfaces for 10 minutes, fixed with 3% PFA and immunolabeled with αphosphotyrosine (*A*). The stimulatory surfaces were prepared using stamps coated with either 25 µg/ml αCD3 (*B & D*); 25 µg/ml αCD28 (*C & G*) or unspecific IgG2a only (*E & F*). The stamped areas were subsequently overlaid with 5 µg/ml αCD28 (*B & F*); 5 µg/ml αCD3 (*C & E*) or unspecific IgG2a only (*D & G*). *B-G*) Top left panels: transmission image; top right panels: CD28-GFP; bottom left: αphosphotyrosine; bottom right panels: overlay of the stamped pattern (blue) and the αphosphotyrosine label (grayscale). In the CD28-GFP and overlay panels the contrast and brightness are adjusted proportionally for clarity. Scale bars 20 µm.

For quantitative immunofluorescence microscopy at the contact site of cells with a surface, variation is prone to arise between different samples due to small differences in focal planes and immunolabeling efficiency. As a consequence, with the analysis of different samples, small but relevant differences in signal intensity between cells or stimuli may be deemed insignificant. In order to overcome this hurdle we developed a protocol to facilitate a comparison of two different cell types on a side-by-side basis (Fig. 2*A*).

Especially in early T cell signal transduction, propagation of the signal is mainly driven through tyrosine phosphorylation [5]. We therefore chose to use phosphotyrosine levels as a marker to assess the impact of CD28 expression levels on early signal initiation. A Jurkat T cell strain with no to low CD28 expression was transfected with CD28-GFP (Fig. S1). After cultivation for two days without selective pressure, the cells were incubated on surfaces functionalized with alternating stripes of αCD3 and αCD28 stimulating antibodies for 10 min. Cells were incubated on surfaces of which the αCD3 stripes were stamped and the αCD28 stripes were overlaid (Fig. 2*B*) and vice versa (Fig. 2*C*) to correct for possible effects of the mode of surface preparation. After fixation, phosphotyrosine levels at the interface of the cells and surfaces were analyzed by confocal laser scanning microscopy using immunofluorescent staining. Labeling controls showed no aspecific clustering of the fluorophores (Fig. S2).The 10-min time point was selected as it provided sufficient time for cell spreading to occur, yet tyrosine microclusters could still be detected all over the cells. In order to sample large numbers of cells we scanned the maximal field of view at a lateral sampling frequency yielding diffraction limited resolution (for an example refer to Fig. S3).

**Figure 3:**
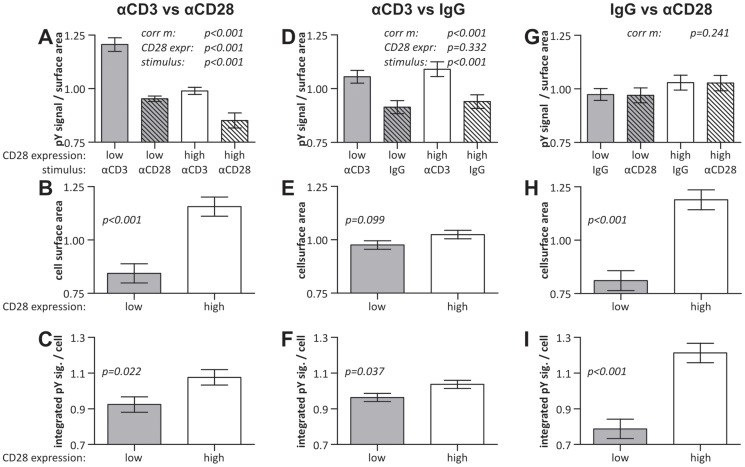
Quantification of the effect of CD28 expression on cell surface spreading and tyrosine phosphorylation. The original images of the experiment of Fig. 2 were quantified (see Macro S1) and the values were normalized to the mean value of the measured property within that image. Normalized values of experiments with inverted stamp and overlay configurations were pooled. The graphs show the mean ± SEM. *A-C*) Cells stimulated with stripes containing αCD3 and stripes containing αCD28. (*n* = 10 images from two separate samples in which stamp and overlay stimuli were reversed (Fig. 2*B & C*) in total counting 1010 CD28 low and 127 CD28 high cells). *D-F*) Cells stimulated with stripes containing αCD3 and stripes containing unspecific IgG2a only. (*n* = 10 images from two separate samples in which stamp and overlay stimuli were reversed (Fig. 2*D & E*) in total counting 921 CD28 low and 97 CD28 high cells). *G-I*) Cells stimulated with stripes containing unspecific IgG2a only and stripes containing αCD28. (*n* = 10 images from two separate samples in which stamp and overlay stimuli were reversed (Fig. 2*F & G*) in total counting 1006 CD28 low and 165 CD28 high cells). *A, D & G*) The background-corrected, αphosphotyrosine intensity per surface area. Corrected model p-values were determined by two-way factorial ANOVAs in which no interaction terms were included. *B, E & H*) The contact surface area per cell. Two-sample T-tests were used to generate the p-values. C, *F & I*) The integrated, background-corrected, αphosphotyrosine intensity per cell (Two-sample T-tests).

When cells were stimulated with parallel stripes of αCD3 and αCD28 a clear accumulation of the CD28 receptor was observed on the αCD28 stripes (Fig. 2*B *& *C*). In contrast the formation of phosphorylated tyrosine clusters primarily took place on αCD3 stripes. Additionally, it appeared that Jurkat T cells expressing high levels of CD28, as judged by GFP intensity (CD28-high cells), covered larger surface areas than CD28-low cells did. The CD28-high cells, however, appeared to have a lower degree of tyrosine phosphorylation than CD28-low cells, both on αCD3 and on αCD28 stripes. In order to verify these observations we quantified the fluorescent intensities (Macro S1). To avoid artifacts as a result of the manner in which the stripes were prepared, the normalized results of both orientations of the experiment (Fig. 2*B *& *C*) were pooled. Data within images was normalized to the mean value within that image in order to eliminate variations between samples and experiments. The protocol yielded unpaired parametric statistical tests and provided information about relative quantitative differences between stimuli and cell types (*Fig. 3*). Datasets for each condition had comparable variances and followed normal distributions.

Quantification showed that cells indeed had a higher degree of tyrosine phosphorylation on αCD3 stripes than on αCD28 stripes (Fig. 3*A*). This effect was independent of CD28 expression levels, meaning that there was no significant difference in the increase between CD28-high and CD28-low cells. Furthermore, it confirmed that, on both αCD3 and αCD28, CD28-high cells had significantly lower phosphotyrosine levels per surface area than CD28-low cells. Expression of CD3 had not been reduced as a consequence of CD28-GFP expression (Fig. S1) and could therefore not have been the cause of this reduced phosphorylation. However, when the local phosphotyrosine densities were corrected for the increased cell spreading (Fig. 3*B*), CD28-high cells seemed to have a slightly higher total tyrosine phosphorylation level, but after a Bonferroni correction this difference could not be shown to be significant (Fig. 3*C*). Without CD28 costimulation (Fig. 2*D *& *E*), no significant differences were found between CD3 stimulated CD28-low and CD28-high cells in the degree of tyrosine phosphorylation per surface area (Fig. 3*D*), interaction surface area per cell (Fig. 3*E*) or total tyrosine phosphorylation per cell (Fig. 3*F*). As expected, significantly higher levels of phosphotyrosine were observed on αCD3 stripes in these samples. It should be noted that this difference was noted primarily on samples where αCD3 was applied as an overlay. When αCD3 was stamped, in many cells phosphotyrosine levels were observed to be higher on the overlay. However, as explained above, we corrected for this effect by pooling data from samples with inversed stamp-overlay orientations.

Finally, when αCD28 stripes were compared with IgG control stripes (Fig. 2*F *& *G*) no significant differences were found in the phosphotyrosine signal per surface area between stripes or cells (Fig. 3*G*). The presence of αCD28 stripes did however stimulate the CD28 cells to form larger interaction surfaces with the stripes (Fig. 3*H*). This indicates that Jurkat T cells can respond to CD28 stimulation alone when high levels of CD28 are expressed, albeit in a limited fashion. The increased surface area of CD28-high cells was accompanied by a proportionate increase in total phosphotyrosine signal per cell (Fig. 3*I*). As we expect this fluorescence to be of membrane proximal, background phosphorylation levels independent of TCR and CD28 signaling, the proportionate increase in total phosphotyrosine signal per cell with increased cell spreading is unsurprising. Even though sample-to-sample variation imposes limits to comparisons between samples, cells clearly responded in a strongly reduced fashion to unspecific IgG2a coated surfaces as compared to αCD3/αCD28 patterned surfaces (Fig. S4). For none of the combinations of stimuli a significant interaction factor between CD28 expression and stimulating surface was found when two-way factorial ANOVAs including interaction terms were applied. Therefore there were no detectable differences in the response to the different stimuli between CD28-low and CD28-high cells. In other words, even though the CD28-low cells had higher local phosphotyrosine signals, the increase of tyrosine phosphorylation on αCD3 was comparable between the two cell types.

Treatment with cytochalasin D, an inhibitor of actin polymerization, drastically reduces cell adherence and spreading (Fig. S5) indicating that Jurkat T cells do not passively adhere to or spread on the striped surfaces and that the observed affects are an effect of CD28 costimulation.

### SHP2 depletion increases cluster phosphorylation but not cluster numbers and decreases IL2 production

The analysis of phosphotyrosine levels, as described above, shows the potential of the striped pattern to perform a side-by-side analysis of two different stimuli. Importantly, we observed distinct differences in tyrosine phosphorylation and surface distribution between Jurkat T cells expressing different levels of CD28. Next, we intended to specifically address the role of the PTP SHP2 in cluster formation and phosphorylation and the CD28 dependence of the observed effects.

SHP2 is one of several PTPs involved in T cell signaling and its effects might therefore be relatively small. Moreover, the protein has been implicated to be involved in both activation and inhibition of cell signaling. By comparing a SHP2 knock-down clone of Jurkat E6.1 (SHP2 KD) with the ‘wild type’ Jurkat E6.1 line (wt) on striped surfaces we wanted to gain insight into whether this phosphatase noticeably affects overall tyrosine phosphorylation. In addition the effect on the tyrosine residue 783 of PLCγ1 in particular was tested as a candidate target of SHP2. In contrast to the combination of stimuli used above, in these experiments we intended to more closely capture the physiological setting of CD28 costimulation in early signaling, which is in colocalization with CD3 engagement. Therefore αCD3+αCD28 mixtures were compared to αCD3 alone.

**Figure 4:**
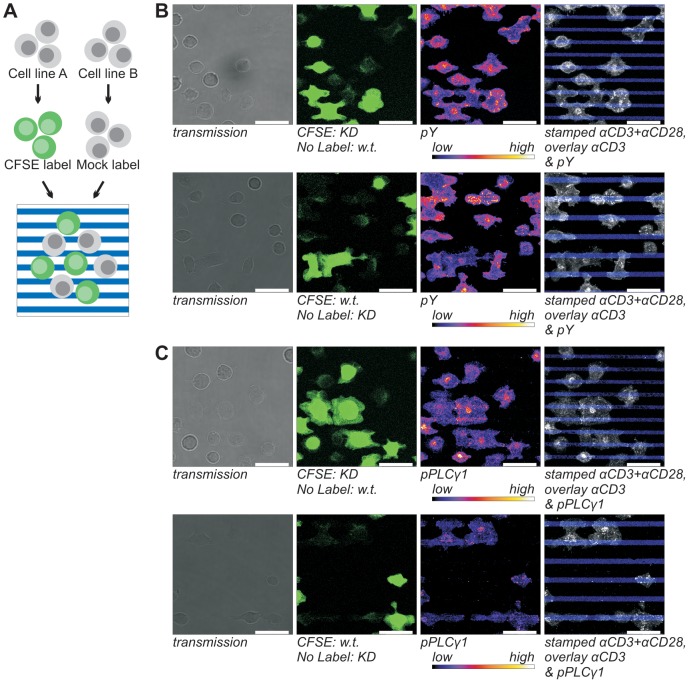
Detection of the stimulus dependence of total tyrosine phosphorylation (*B*) and phosphoY783 PLCγ1 (*C*) in Jurkat cells and SHP2 KD cells. *A*) For the side-by-side analysis of signaling in Wt and SHP2 KD Jurkat E6.1 T cells, one of the lines was labeled with the cell tracer CFSE. After overnight serum starvation the cells are pooled and incubated on micropatterned, stimulating surfaces for 10 min. Subsequently, the cells are fixed with 3% PFA, permeabilized and immunolabeled for the detection of signaling clusters. *B & C*) In the top panels, SHP2 KD cells are CFSE labeled and in the bottom panels, wt cells are labeled. Panels from left to right: transmission images; CFSE; immunofluorescence; overlay of the stamped pattern (blue) and the immunolabel (grayscale). In the overlay panels the contrast and brightness for both channels were adjusted proportionally for clarity. 12.5 µg/ml αCD3 + 12.5 µg/ml αCD28 coated stamps were used to generate a striped pattern which was overlaid with 5 µg/ml αCD3. CFSE channels were recorded with saturated signals to facilitate image processing. Scale bars 20 µm.

In Jurkat E6.1 SHP2 KD cells the phosphatase was down-regulated by expression of lentivirally transduced shRNA. In comparison to wt cells, SHP2 expression was reduced to 13% in these cells (Fig. S6 *A*), but this had no effect on receptor expression (Fig. S6 *B* & *C*). SHP2 KD and wt Jurkat cells were incubated on stripes functionalized with a 1:1 ratio of αCD3 and αCD28 alternating with stripes of only αCD3 for 10 min and stained for phosphotyrosine or phosphoY783 PLCγ1. By labeling one of two cell types with the cell tracer CFSE prior to incubation on micropatterned surfaces (Fig. 4*A*) the two types could easily be distinguished during microscopy (Fig. S3). We confirmed that all CFDA-SE treated cells were fluorescently labeled (Fig. S7).

Again confocal images were acquired with the focus on the plane of the contact area. Both cell lines responded in a comparable heterogeneous fashion to the stripes (Fig. S3). For both Jurkat strains approximately 80% of the cells had formed microclusters of pY or pPLCγ1 and most cells had higher cluster numbers and increased phosphotyrosine (Fig. 4*B*) and pY783 PLCγ1 signals (Fig. 4*C*) on the stripes containing both stimuli. However, some cells also formed large numbers of clusters on the αCD3 coated surface. Interestingly, the cluster brightness varied strongly between cells within images. Additionally, cells spread more on stripes containing both stimuli than on stripes consisting of only αCD3 (Fig. 4*B *& *C*). This contact difference was less pronounced when αCD3 was stamped and αCD3+αCD28 was overlaid (Fig. S3, S4 & S7), indicating that, as above, stamping resulted in a different activity of the stimuli than functionalization by incubation with soluble antibodies. Therefore, experiments were also performed in which the stamped and overlaid stimuli were switched (results not shown but included in the quantitative analyses below). Comparable results were obtained independent of which cell strain was CFSE labeled (compare top and bottom panels of Fig. 4*B* & *C*).

**Figure 5:**
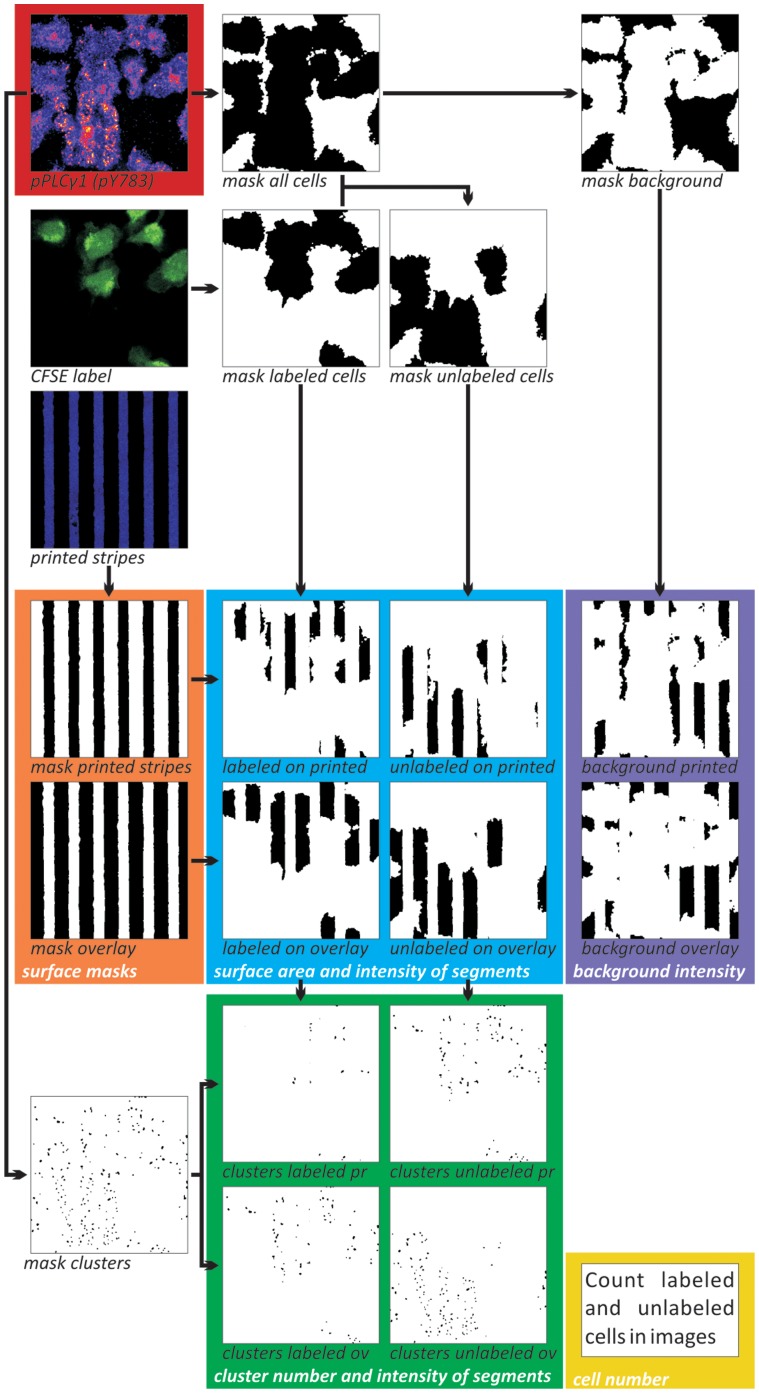
Image processing of phosphoPLCγ1 signals and cluster formation. Overview of the image processing protocol as described in Materials and Methods and used for the analysis of the experiments described in Fig. 4. In order to resolve clusters in print, an enlarged segment of a microscopy image labeled with αphospho-PLCγ1 (Fig. S3) is shown as an example. Image processing and quantification was done on a per image basis. Macro S2 describes the full procedure utilized to analyze the images. In short, the pPLCγ1 signal was thresholded to generate a binary mask of all cells. This image was inverted to generate a mask of the background signal. The CFSE image was thresholded and was used in combination with the mask of all cells to generate a mask of CFSE labeled cells and a mask of unlabeled cells. The image of the printed stripes was thresholded to generate a mask of the printed structures and inversed to also generate a mask of the overlaid areas. Combining the masks of the printed structures and overlaid areas with the masks of the cells formed the masks of the CFSE labeled cells on stamped stripes, the CFSE labeled cells on overlaid structures, the unlabeled cells on stamped stripes and the unlabeled cells on overlaid structures. These four masks were used to measure the surface areas the cells covered on both surfaces. Combining the stripe and overlay masks with the background mask enabled the measurement of surface areas not covered by cells. The last six generated masks were, in turn, applied to the original pPLCγ1 image and from the resulting images the total pPLCγ1 signal per condition could be determined. Together with the total surface areas of the specific condition, the signal intensity per µm^2^ was calculated. Surface specific background corrections were applied. In addition, a binary cluster mask was generated from the pPLCγ1 image. This mask was segmented using the four masks of cells on surfaces creating four new masks. From these masks cluster numbers were counted and by applying them to the original pPLCγ1 image cluster intensities could be determined. Finally, the cell numbers per image were determined by eye using the original transmission images and the cell masks. The various colors correspond to the graphs in Fig. 6 and indicate which masks and images are required to produce the particular data.

Due to the heterogeneity of the cell response, quantitative analyses were necessary to extract subtle differences between SHP2 KD cells and the wt Jurkat cells. For this purpose we extended our image processing protocol for extensive quantification of clusters and cell surface distribution (Macro S2 & Fig. 5).

As before, the normalized values of multiple images of several experiments, in which the orientation of stamped and overlaid surface and CFSE labeled and unlabeled cells varied, were pooled. For each condition, datasets followed normal distributions and groups showed comparable variances.

**Figure 6:**
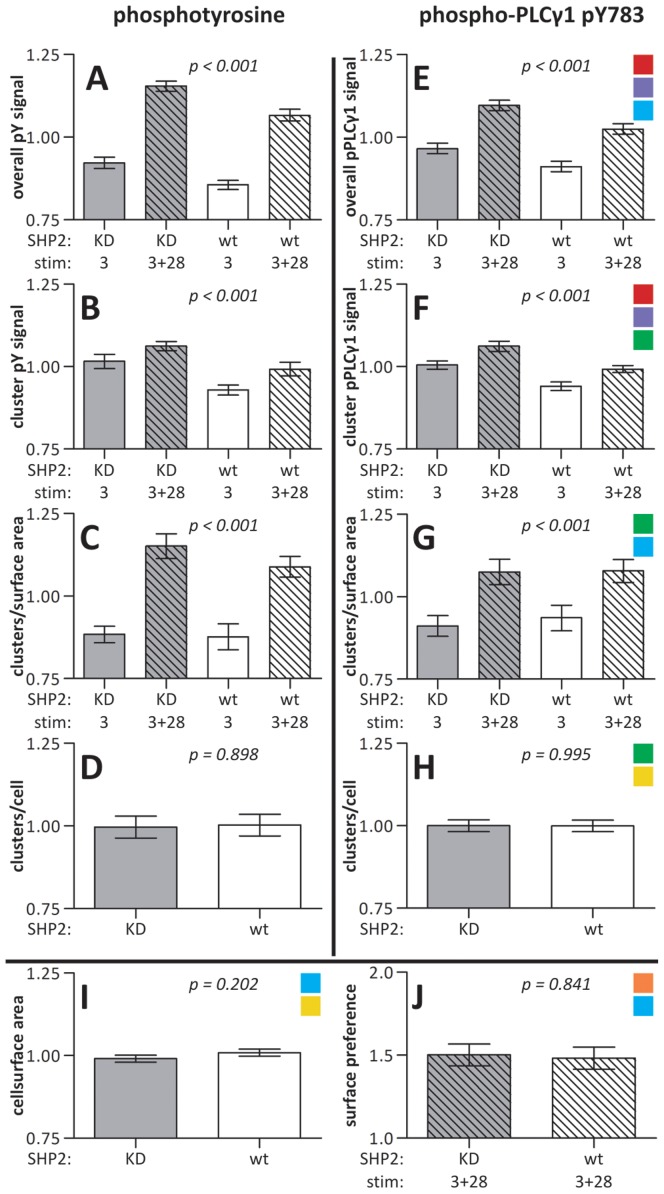
Quantification of the effects of CD28 costimulation and SHP2 deficiency. The values acquired through image segmentation as described in Fig. 5 were normalized to the mean value of the specific property for that image. The information of multiple images from multiple experiments was used for further analyses. The graphs depict the stimulus and SHP2 dependence of spreading and tyrosine phosphorylation showing the mean ± SEM (based on number of images) of the respective property. KD  =  SHP2 knock-down E6.1 Jurkat cells; wt  =  wild type E6.1 Jurkat cells; 3  =  stripes of αCD3 alone; 3+28  =  αCD3+αCD28-containing stripes (Fig. 4). The colored squares correspond to the colors bordering images and masks in Fig. 5 used to retrieve the data required for the graph in question. Corrected model p-values were determined by two-way factorial ANOVAs in which no interaction terms were included (*A-C & E-G*) or two-sample T-tests (D *& H-J*). *A-D*) Cells labeled with the αphosphotyrosine antibody (*n* = 15 images resulting from three separate experiments with varying CFSE/unlabeled and stamp/overlay conditions in total containing 861 KD and 615 wt cells). *E-H*) Cells labeled with the αphosphoY783-PLCγ1 antibody (*n* = 26 images resulting from five separate experiments with varying CFSE/unlabeled and stamp/overlay conditions in total containing 1804 KD and 1502 wt cells). *A & E*) Average, background-corrected, overall intensity per surface area. *B & F*) Average, background-corrected intensity of cluster pixels. *C & G*) Average number of clusters per surface area. *D & H*) Average number of clusters per cell. *I & J*) The average contact surface area per cell (*I*) and surface-preference-score (*J*, see text) were determined from pooled data from the phosphoTyr and phosphoY783 PLCγ1 experiments (*n* = 41 images from 8 experiments with varying CFSE/unlabeled and stamp/overlay conditions in total containing 2665 KD and 2117 wt cells).

**Figure 7:**
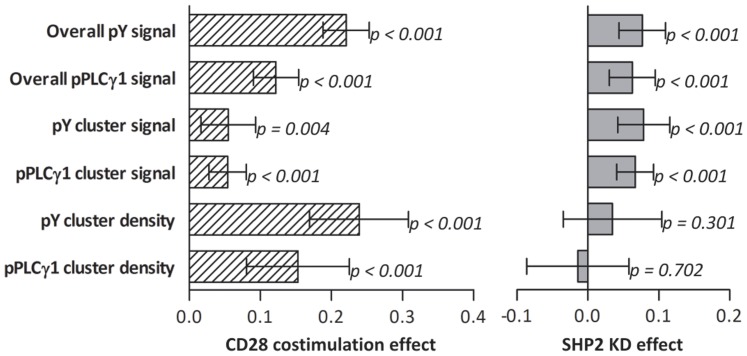
Impact of CD28 costimulation and SHP2 deficiency on cluster numbers and phosphorylation. Effects with 95% confidence intervals of CD28 costimulation (left) and the knock down of SHP2 (right) as predicted by ANOVAs on properties for which significant differences were found in Fig. 6. The effect is given as a fraction of the overall mean value for that specific property.

Quantification of the images revealed small but significant differences in early signaling events between SHP2 KD and wt Jurkat T cells. SHP2 KD cells had a 7.7% higher phosphotyrosine signal than wt cells (95% confidence interval (CI) 4.5%–10.9%; Fig. 6*A* & Fig. 7). In parallel the intensity of the phosphorylated tyrosine microclusters was 7.9% higher in these cells (CI 4.3%–11.5%; Fig. 6*B* & Fig. 7). Similarly, the specific phosphorylation of tyrosine residue 783 in PLCγ1 was 6.3% higher (CI 3.2%–9.4%; Fig. 6*E* & Fig. 7) as was the cluster-specific intensity (6.7%, CI 4.1%–9.3%; Fig. 6*F* & Fig. 7) in cells not expressing SHP2. There were no significant differences between the cell strains in the number of microclusters (Fig. 6*C, D, G, H* & Fig. 7), cell size (Fig. 6*I*) or surface preference (Fig. 6*J*; see below). See *Table 1* for absolute values.

**Table 1:** Measured cluster numbers and cell sizes.

Property	SHP2 KD		wt	
pY clusters per cell	15.1±2.07		15.8±2.27	
pPLCγ1 (pY783) clusters per cell	12.9±0.77		13.0±0.88	
cell contact surface (µm^2^)	167±3.93		170±4.24	
	**KD 3**	**KD 3+28**	**wt 3**	**wt 3+28**
pY clusters per 100 µm^2^	8.9±0.97	11.7±1.39	9.2±1.17	11.4±1.50
pPLCγ1 (pY783) clusters per 100 µm^2^	7.8±0.43	9.6±0.73	8.0±0.52	9.6±0.68

Values are given as mean ± SEM. KD =  SHP2 knock-down E6.1 Jurkat cells; wt =  wild type E6.1 Jurkat cells; 3 =  αCD3 stimulus alone; 3+28 =  αCD3+αCD28-containing stripes.

In addition to the effects of SHP2 deficiency, there were also clear differences between αCD3 stimulation alone and αCD3+αCD28 costimulation. Cells formed 23.9% more phosphotyrosine microclusters per µm^2^ on stripes of mixed stimuli than on stripes of only αCD3 (CI 17.2%–30.7%; Fig. 6*C* & Fig. 7). Also, the density of phosphorylated PLC1γ1 microclusters was higher on αCD3+αCD28 than on αCD3 surfaces (15.3%, CI 8.3%–22.4%; Fig. 6*G* & Fig. 7). The variance of the absolute number of signaling clusters per surface between images was much larger than the one of the normalized figures and therefore did not give significant information (Table 1).

This higher cluster density on αCD3+αCD28 coated surfaces is reflected in the overall signal intensities of the cells on the different surfaces. For phosphotyrosine this signal was 22.1% higher on αCD3+αCD28 stripes than on αCD3 stripes (CI 18.9%–25.3%; Fig. 6*A* & Fig. 7). The 5.5% intensity increase of the clusters on mixed surfaces contributes relatively little to the large overall increase (CI 1.9%–9.1%; Fig. 6*B* & Fig. 7). For phosphoPLCγ1 the overall signal was 12.2% higher (CI 9.1%–15.3%; Fig. 6*E* & Fig. 7) and the microclusters were 5.4% more intense (CI 2.8%–8.0%; Fig. 6*F* & Fig. 7).

After having determined a direct effect of CD28 expression on cell spreading we aimed to assess in more detail the effect of CD28 costimulation on membrane distribution and spreading. In order to quantify the preference of cells for contacting one of the two surfaces we devised a surface-preference-score (Fig. 6*J*, eq. 1). The score for the αCD3+αCD28 surface is defined as the ratio of cell surface on αCD3+αCD28 over cell surface on αCD3 stripes corrected by the ratio of the total αCD3+αCD28 surface over the total αCD3 surface.
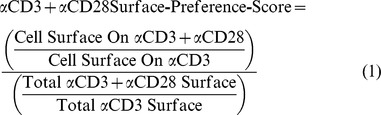



A surface-preference-score of 1 indicates no preference, a score > 1 indicates a preference for the cells to establish contact with αCD3+αCD28 and a score < 1 indicates that the cells prefer αCD3 surfaces. Both cell strains had a clear preference for the αCD3+αCD28 surface as determined by one-sample T tests (p<0.001 for both; test value  =  1). Together with the observed stretched shapes of the cells (Fig. S3 & Fig. 4) this clearly demonstrates that CD28 engagement also increases cell spreading in a costimulatory setting. No difference in surface preference was found between SHP2 KD and wt cells (Fig. 6*J*).

As before, no significant interaction factors between cell type and stimulating surface were found, indicating that there is no detectable difference in the effect of CD28 costimulation between wt and SHP2 KD cells.

**Figure 8:**
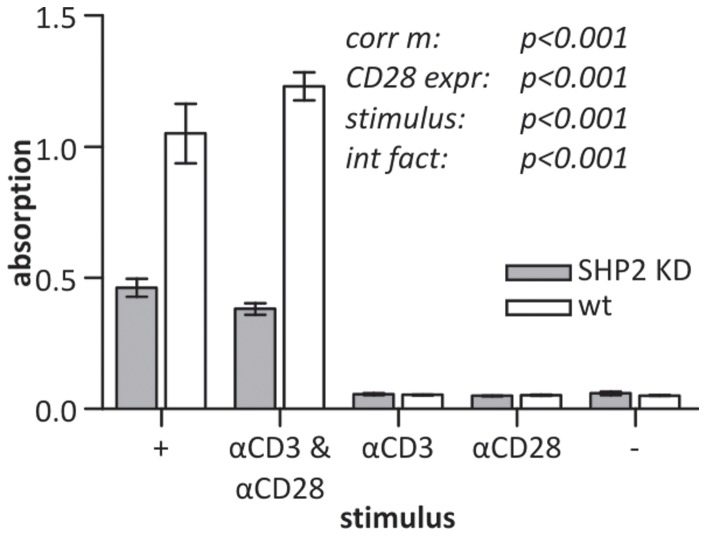
Effect of SHP2 depletion on IL2 expression. SHP2 KD and wt Jurkat E6.1 T cells were stimulated with PMA + ionomycin (+), αCD3 & αCD28, αCD3 alone, αCD28 alone or were left unstimulated (–) for 22 h. IL2 in the supernatants was quantified by sandwich ELISAs. Given are the absorption values ± SEM. The p-values are from a full factorial two-way ANOVA and represent the significance of the overall corrected model (corr m), the effect of CD28 expression (CD28 expr), the effect of the stimulus and the interaction factor (int fact) between stimuli and CD28 expression. For all conditions *n* = 3 samples, all from a single experiment representative of four independent experiments.

After having found that the inhibition of SHP2 expression stimulates the early T cell signaling response by increasing pY and pPLCγ1, we probed for the induction of IL2 expression to address whether late T cell responses were also affected. SHP2 KD cells had a significantly reduced production of IL2 when stimulated with αCD3 and αCD28 compared to wt cells (Fig. 8). This effect was not restricted to extracellular stimulation but was also observed when PMA and ionomycin were used. This difference is remarkably different from the positive impact of SHP2 deficiency on early tyrosine phosphorylation. A Bonferroni post-hoc test showed that there were no significant differences between cells stimulated with PMA + ionomycin and cells stimulated with αCD3 + αCD28. One may argue that the difference in IL2 production observed is due to stimulation-dependent apoptosis. However, levels of apoptosis were not found to be different for wt versus SHP2 KD cells, indicating that the observed difference could be attributed to an actual reduced IL2 production per cell (Fig. S8).

## Discussion

Protein cluster formation is a hallmark of early T cell signaling and has received significant attention. Studies have addressed the effect of pMHC engagement, cluster migration, localization and colocalization of microclusters of many different signaling proteins over time [11], [17], [30], [31], [53], [54], [55], [56]. Recently, photo-activatable localization microscopy and direct stochastic optical reconstruction microscopy have been used for a detailed, quantitative analysis of LAT clusters and their phosphorylation at resolutions down to 20 nm [57], [58].

Here, we established microcontact printing in combination with image processing for a quantitative analysis of stimulus-dependent protein microcluster formation in early T cell signaling. In a first step, we established that different levels of CD28 expression translated into different responses on antibody-coated surfaces. Consistent with a positive stimulatory role in signaling, Jurkat T cells expressing high levels of CD28 covered larger surface areas than CD28-low cells when stimulated with parallel stripes of αCD28 and αCD3 or combinations of αCD28 and IgG control stripes. Interestingly, we were not able to detect an increased level of tyrosine phosphorylation in CD28-high cells. When no CD28 costimulus was present, no significant difference between the two cell lines was observed. This indicates that CD28-GFP expressing cells had not been compromised in their potential for activation through the stimulation of CD3. It has been shown that CD4^+^ T cells of rheumatoid arthritis patients express higher levels of CD28 and other markers of activated T cells than those of healthy controls [59]. The protocol presented here can serve as a tool to study how early signaling in such aberrant cells is affected and possibly provide clues for suitable treatments.

By performing a detailed side-by-side quantitative analysis of phosphotyrosine clusters on αCD3 and αCD3+αCD28 coated surfaces, we addressed to which extent the number and intensity of clusters were a function of the stimulus and the presence of an individual signaling protein.

CD28 costimulation led cells to form an increased density of phosphorylated microclusters (24% for pY and 15% for pY783 PLCγ1) and relatively small increases in phosphotyrosine intensity of the clusters. Additionally, αCD3+αCD28 induced stronger local spreading than αCD3 alone. These results and the results discussed above show that CD28 plays a significant role in spreading of T cells suggesting that CD28 stimulation induces a T cells to more thoroughly probe the surface or APC it is currently engaging, even in the absence of CD3 engagement. Costimulation of T cells with CD28 has been previously demonstrated to promote expression of proteins involved in cytoskeletal remodeling [60] and the CD28 signal invokes actin reorganization and formation of lamellipodia through PI3K [21], cofilin [61] and Rho family GTPases [62]. Our data supports the notion that CD28 costimulation initiates qualitatively different signaling pathways than stimulation of the TCR.

The impact of SHP2 deficiency on cluster formation was qualitatively and quantitatively different from the impact of costimulation. In contrast to the effect of CD28 engagement, no significant difference in phosphorylated cluster density was observed. However, SHP2 deficiency did lead to a small but significant increase of overall and cluster tyrosine phosphorylation and PLCγ1 Y783 phosphorylation. PTP activity greatly exceeds kinase activity [63] and other PTPs may have overlapping substrate specificity with SHP2. Nevertheless, knock down of this single phosphatase had a perceivable effect on overall phosphotyrosine levels. This demonstrates that the loss of SHP2 cannot be fully compensated by other phosphatases, such as SHP1, and therefore plays a non redundant role in T cell signaling. Interestingly, it has been recently found by Yokosuka et al. [44] that upon stimulation of the TCR and the negative regulator programmed cell death 1 (PD1), SHP2 itself forms clusters. In T cells expressing a phosphatase-dead dominant-negative form of SHP2 the phosphorylation of PD1 was increased which is in line with our observation of increased tyrosine phosphorylation.

In summary, these observations demonstrate that CD28 engagement contributes to the formation of clusters acting as signaling platforms, while SHP2 targets already formed signaling clusters. There were no indications that SHP2 specifically targets CD28 signaling.

Interestingly, for late T cell activity a reversed and large effect of SHP2 deficiency was observed. While general phosphotyrosine and phospho-PLCγ1 signals were higher in the SHP2 KD cells during early signaling, IL2 production was lower as described previously [45]. This means that higher tyrosine phosphorylation levels during the first ten minutes of T cell stimulation do not necessarily result in a stronger T cell response. It also shows that SHP2, despite being one of many PTPs in T cells, has a significant regulatory effect on T cell activation. CD3 and CD28 stimulation were both necessary to generate an IL2 response. IL2 expression was also reduced for cells stimulated with PMA and ionomycin suggesting that SHP2 exerts this latter effect at a later stage of the signaling cascade than the initial dephosphorylating effect on PLCγ. The effect on cytokine secretion observed is likely due to the positive effect of SHP2 on MAPK signaling [45], [46] which is crucial for IL2 production [64]. Further research, however, is required in order to verify this hypothesis. Remarkably, it appears that SHP2 plays a dual role in IL2 production as Yokosuka et al. [44] observed SHP2, through PD1, negatively affected IL2 production.

The combination of micropatterned surfaces with quantitative image processing as demonstrated here, adds a valuable and accessible tool to the repertoire of analytical techniques in the analysis of early T cell signaling. Image processing is applied to a cell population in an unbiased fashion. The stamping of stripes enables a highly sensitive side-by-side analysis of different stimuli on a microscale level, which can be further extended to a side-by-side comparison of different cell strains eliminating noise arising from sample-to sample variation. Even though state-of-the-art superresolution techniques provide the means to visualize single molecules within clusters, challenges such as cell-to-cell and sample-to-sample variation still apply to these more advanced techniques.

In this study we addressed the role of the PTP SHP2 in cluster formation and phosphorylation using a SHP2 KD Jurkat strain next to wt Jurkat cells. However, quantitative comparisons of signaling can benefit the analysis of T cell biology in multiple other ways. T effector cells and T regulatory cells, for example, show very limited differences in the expression of signaling proteins, yet widely differ in their physiological role [65]. The approach shown here can be of great benefit to the quantitative understanding of the functional implications of differences in early T cell signaling.

## Supporting Information

Figure S1 **Over-expression of CD28 does not affect CD3 expression.** Expression levels of CD28 (middle row) and CD3 (bottom row) were determined with flow cytometry for non-transfected Jurkat T cells (ACC-282; left) and CD28-GFP transfected cells (right). The top row shows a negative control in which cells were treated with unspecific IgG2a. Scatter plots with GFP expression on the X-axis and the immunolabelled receptors (Zenon Alexa 647) on the Y-axis are depicted.
(TIF)

Figure S2 **Phospho tyrosine and phospho-PLCγ1 labelling control.** Jurkat T cells were serum starved overnight and incubated on striped surfaces for 10 minutes. Surfaces were functionalized using stamps coated with 25 µg/ml αCD3 and overlaid with 2.5 µg/ml αCD3 + 2.5 µg/ml αCD28. Samples were immunolabeled with αphosphotyrosine conjugated with Zenon Alexa Fluor 546 component A and blocked with component B (*A*), the Zenon Alexa Fluor 546 component A blocked with component B without specific antibody (*B*), phosphoY783 PLCγ1 and αrabbit Alexa Fluor 546 (*C*) or αrabbit Alexa Fluor 546 only (*D*). Images were acquired with a Zeiss LSM510 meta confocal laser scanning microscope using a 63×1.4 N.A. PLAN APO objective and 543 nm and 633 nm HeNe lasers (Carl Zeiss, Sliedrecht, The Netherlands). Left panels: immunolabel. Right panels: stamped patterns. Contrast and brightness were adjusted proportionally. Scale bars 5 µm.
(TIF)

Figure S3 **Overlay of typical microscopy images used for analysis.** One field of view at 2048 × 2048 pixels. In this case stamps coated with 25 µg/ml αCD3 were used to generate a striped pattern (blue) which was overlaid with 2.5 µg/ml αCD3 + 2.5 µg/ml αCD28. The CFSE labeled (green) SHP2 KD Jurkat T cells are clearly distinguishable from the non-CFSE labeled wt Jurkat cells. After fixation with 3% PFA the cells were immunolabeled with αphospho-PLCγ1 (grayscale). For clarity, contrast and brightness are adjusted proportionally. Scale bar main image 50 µm; scale bar enlargement 10 µm.
(TIF)

Figure S4 **Tyrosine phosphorylation on control surfaces.** CD28-GFP transfected Jurkat ACC-282 T cells were serum starved for 6 h and then incubated on striped surfaces for 10 minutes, fixed with 3% PFA and immunolabeled with αphosphotyrosine. Surfaces were functionalized using stamps coated with 25 µg/ml αCD3 (*A*) or unspecific IgG2a only (*B*). The remainder was subsequently overlaid with either 5 µg/ml αCD28 (*A*) or unspecific IgG2a only (*B*). Top left panels: transmission image; top right panels: CD28-GFP; bottom left: αphosphotyrosine; bottom right panels: overlay of the stamped pattern (blue) and the αphosphotyrosine label (grayscale). For a better comparison no adjustments were made to the contrast or brightness of the images. Scale bars 50 µm.
(TIF)

Figure S5 **Reduced adherence and spreading of cells treated with cytochalasine D.** Jurkat T cells were serum starved overnight and were treated with 10 µM cytochalasine D (Tocris Bioscience, Bristol, UK) 10 minutes prior to, and during incubation on striped surfaces. Surfaces were functionalized using stamps coated with 25 µg/ml αCD3 and overlaid with 2.5 µg/ml αCD3 + 2.5 µg/ml αCD28. Samples were immunolabeled with αphosphotyrosine. Images were acquired with a Zeiss LSM510 meta confocal laser scanning microscope using a 63×1.4 N.A. PLAN APO objective and 543 nm and 633 nm HeNe lasers (Carl Zeiss, Sliedrecht, The Netherlands). Panels from left to right: transmission image, immunolabel and stamped patterns. Scale bars 20 µm.
(TIF)

Figure S6 **SHP2 expression in SHP2 knock-down cells is reduced to 13% of wild type levels but both lines express receptors at comparable levels.** *A*) Total cell lysates of Jurkat E6.1 SHP2 KD cells and Jurkat E6.1 ‘wt’ cells were subjected to SDS-PAGE followed by immunoblotting of SHP2 expression using a SHP2 antibody (rabbit polyclonal, N-10) from Santa Cruz Biotechnology (Heidelberg, Germany) or β-actin antibodies (mouse monoclonal, AC-15, Sigma-Aldrich, Deisenhofen, Germany). After subsequent incubation with horseradish peroxidase–conjugated secondary antibodies, the blots were developed using Western Lightning chemiluminescence detection (Perkin Elmer Life Sciences, Boston, MA, USA) and quantitatively evaluated using a CCD camera-based system (LAS3000; Fujifilm, Düsseldorf, Germany). SHP2 levels were quantified in relation to β-actin levels. Below, SHP2 expression levels are given relative to levels in wt cells. *B & C*) Expression levels of CD3 (left panels, Zenon Alexa 488) and CD28 (right panels, Zenon Alexa 647) were determined with flow cytometry for SHP2 KD cells (*A*) and wt cells (*B*). The unfilled histograms show isotype controls while the filled histograms αCD3 and αCD28 labeled populations, respectively.
(TIF)

Figure S7 **CFSE fluorescence (green) is retained by all cells after fixation, permeabilization and immunolabeling.** Stamps coated with 25 µg/ml αCD3 were used to generate striped patterns (blue) which were overlaid with 2.5 µg/ml αCD3 + 2.5 µg/ml αCD28. Jurkat E6.1 ‘wild type’ cells were labeled with CFDA-SE (*A*) or mock labeled (*B*), serum starved over night and subsequently incubated on the micropatterned surfaces for 10 minutes, fixed with 3% PFA and immunolabeled with αphospho-PLCγ1 (grayscale). *A* & *B* were recorded with identical microscopy settings and all three channels are overlaid for both. For clarity, contrast and brightness were adjusted proportionally. Scale bar 50 µm.
(TIF)

Figure S8 **SHP2 knock down effect on phosphatidylserine exposure.** Wells of a 96-well flat bottom plate were coated as described for the ELISA in the Materials and Methods section. In these wells 1•10^5^ SHP2 KD or wt Jurkat T cells were stimulated with αCD3 & αCD28 (clone CD28.2; eBioscience, Frankfurt, Germany), αCD3 alone, αCD28 alone or were left unstimulated (-) for 24 (left) or 48 hours (right) at 37°C, 5% CO_2_ and under humidified conditions. Cells were subsequently stained with the Annexin V-PE 7-AAD Apoptosis Detection Kit I (BD Pharmingen, Heidelberg, Germany) using the suppliers protocol. Phosphatidylserine exposure was determined using a FACS Canto flow cytometer (BD Biosciences, Heidelberg, Germany) and characterizing 1•10^4^ cells per sample. The graph shows the percentage of annexin V negative cells ± SEM of three independent experiments.
(TIF)

Macro S1 **Macro used for data extraction from images of CD28-GFP transfected cells exposed to stripes of different stimuli.** This self-written macro was used in combination with ImageJ to analyze the confocal images described in Fig. 2. The macro separates CD28-low and CD28-high cells on the different stripes. Guidelines to determine threshold values are included in the macro.
(TXT)

Macro S2 **Macro used for the cluster analyses in images of CFSE labeled and unlabeled cells on two different types of stimuli.** This self-written macro was used in combination with ImageJ to analyze confocal images described in Fig. 4. of samples generated as described in Materials and Methods. The macro performs segmentation into CFSE labeled and unlabelled cells and signaling clusters on the different stripes as illustrated in Fig. 5. Guidelines to determine threshold values are included in the macro.
(TXT)
