# Supplementary figures and images for: A Quantitative Assessment of Costimulation and Phosphatase Activity on Microclusters in Early T Cell Signaling
Source: PLoS One. 2013 Oct 30;8(10):e79277. doi: 10.1371/journal.pone.0079277 (PMC3813591; doi:10.1371/journal.pone.0079277)

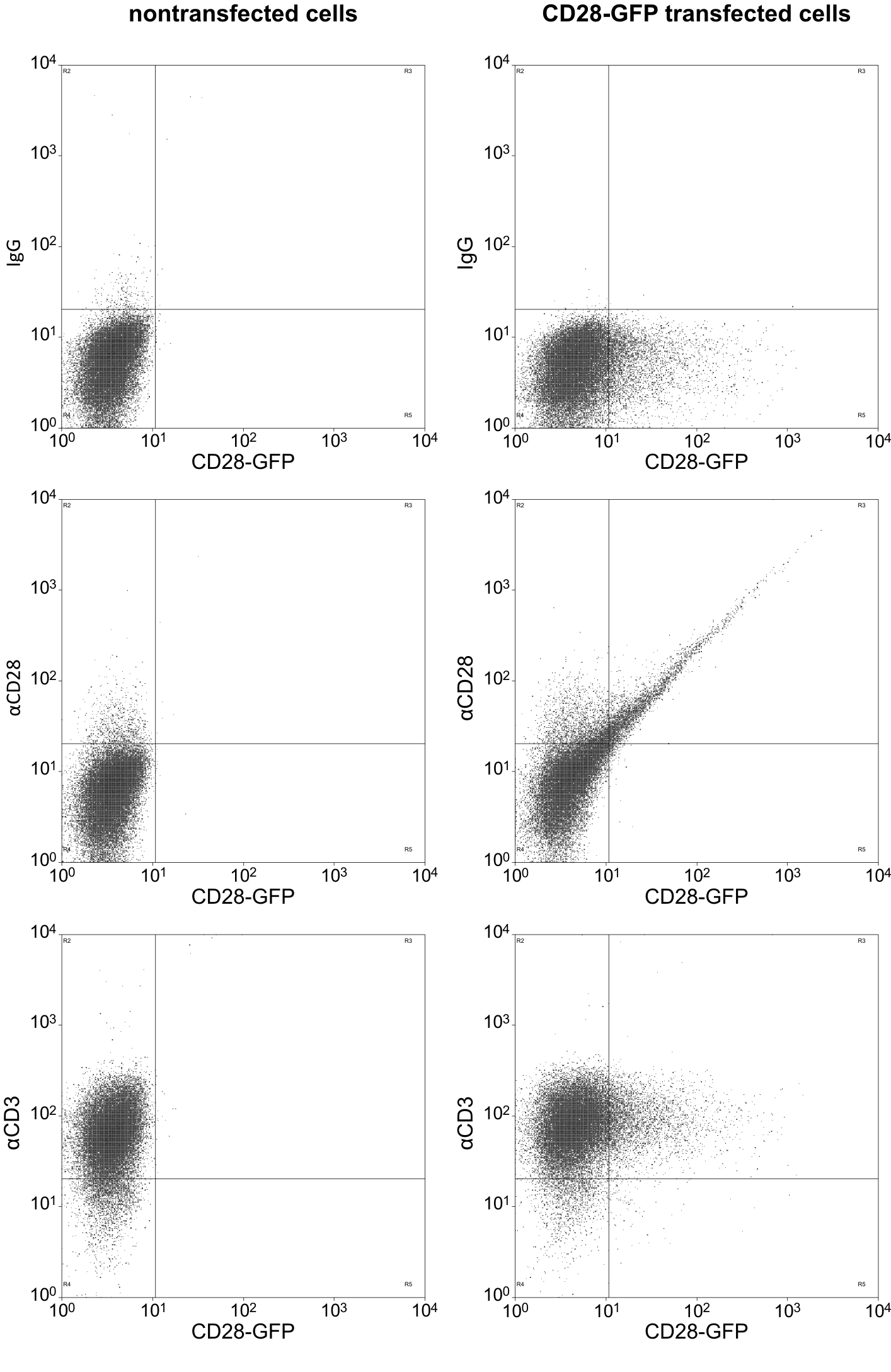

Supplement: Figure S1 — Over-expression of CD28 does not affect CD3 expression. Expression levels of CD28 (middle row) and CD3 (bottom row) were determined with flow cytometry for non-transfected Jurkat T cells (ACC-282; left) and CD28-GFP transfected cells (right). The top row shows a negative control in which cells were treated with unspecific IgG2a. Scatter plots with GFP expression on the X-axis and the immunolabelled receptors (Zenon Alexa 647) on the Y-axis are depicted. (TIF) [file pone.0079277.s001.tif]

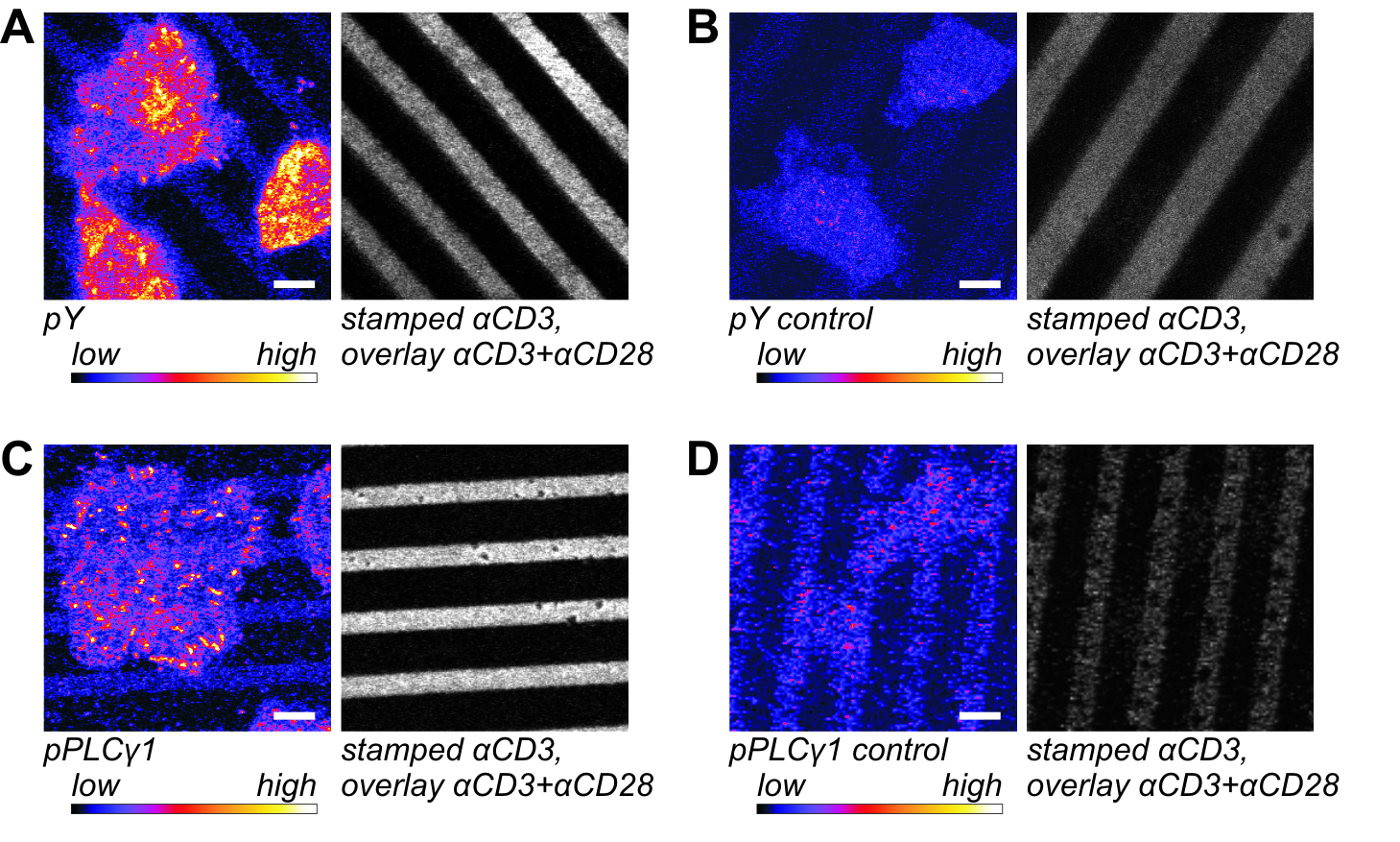

Supplement: Figure S2 — Phospho tyrosine and phospho-PLCγ1 labelling control. Jurkat T cells were serum starved overnight and incubated on striped surfaces for 10 minutes. Surfaces were functionalized using stamps coated with 25 µg/ml αCD3 and overlaid with 2.5 µg/ml αCD3 + 2.5 µg/ml αCD28. Samples were immunolabeled with αphosphotyrosine conjugated with Zenon Alexa Fluor 546 component A and blocked with component B (A), the Zenon Alexa Fluor 546 component A blocked with component B without specific antibody (B), phosphoY783 PLCγ1 and αrabbit Alexa Fluor 546 (C) or αrabbit Alexa Fluor 546 only (D). Images were acquired with a Zeiss LSM510 meta confocal laser scanning microscope using a 63×1.4 N.A. PLAN APO objective and 543 nm and 633 nm HeNe lasers (Carl Zeiss, Sliedrecht, The Netherlands). Left panels: immunolabel. Right panels: stamped patterns. Contrast and brightness were adjusted proportionally. Scale bars 5 µm. (TIF) [file pone.0079277.s002.tif]

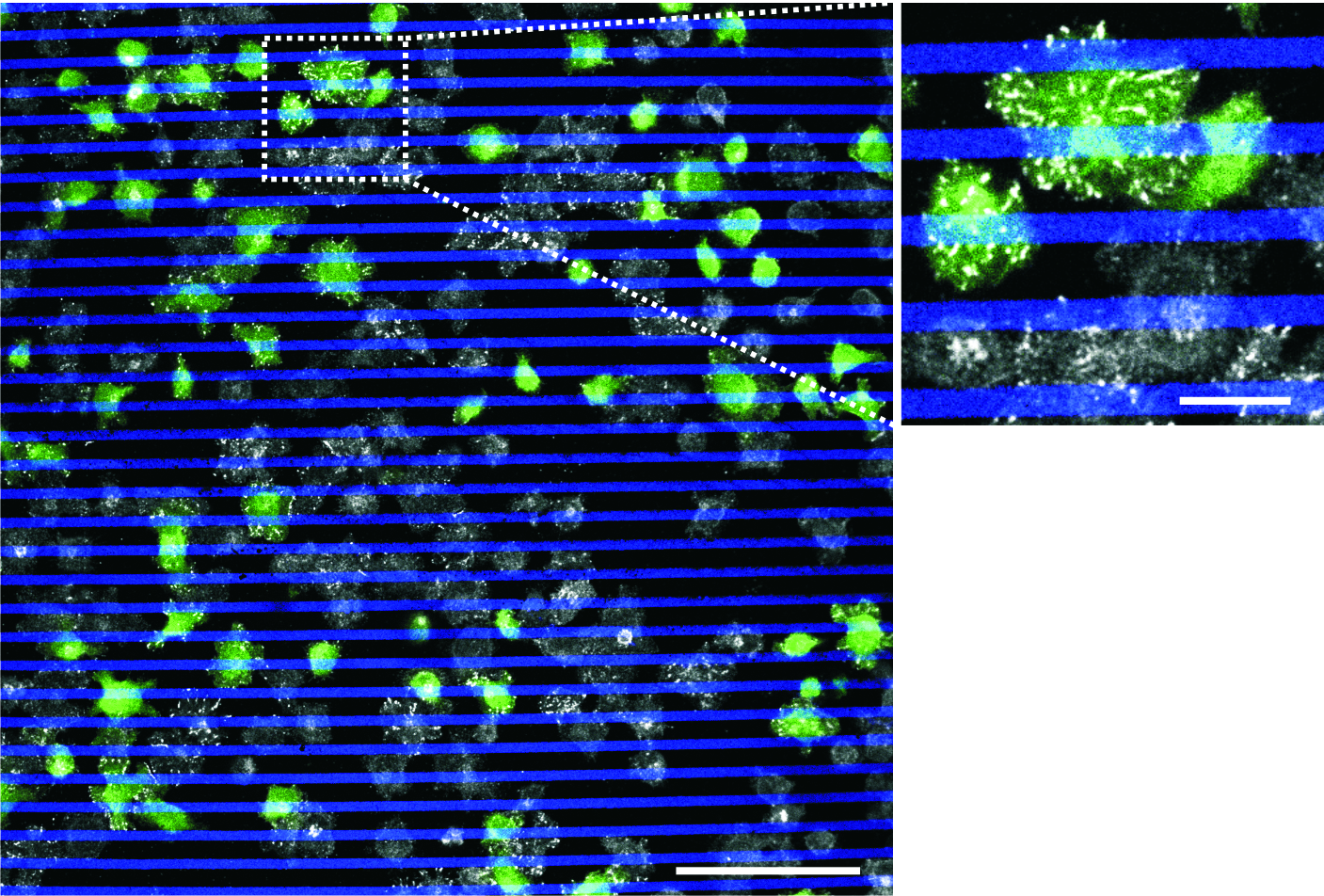

Supplement: Figure S3 — Overlay of typical microscopy images used for analysis. One field of view at 2048 × 2048 pixels. In this case stamps coated with 25 µg/ml αCD3 were used to generate a striped pattern (blue) which was overlaid with 2.5 µg/ml αCD3 + 2.5 µg/ml αCD28. The CFSE labeled (green) SHP2 KD Jurkat T cells are clearly distinguishable from the non-CFSE labeled wt Jurkat cells. After fixation with 3% PFA the cells were immunolabeled with αphospho-PLCγ1 (grayscale). For clarity, contrast and brightness are adjusted proportionally. Scale bar main image 50 µm; scale bar enlargement 10 µm. (TIF) [file pone.0079277.s003.tif]

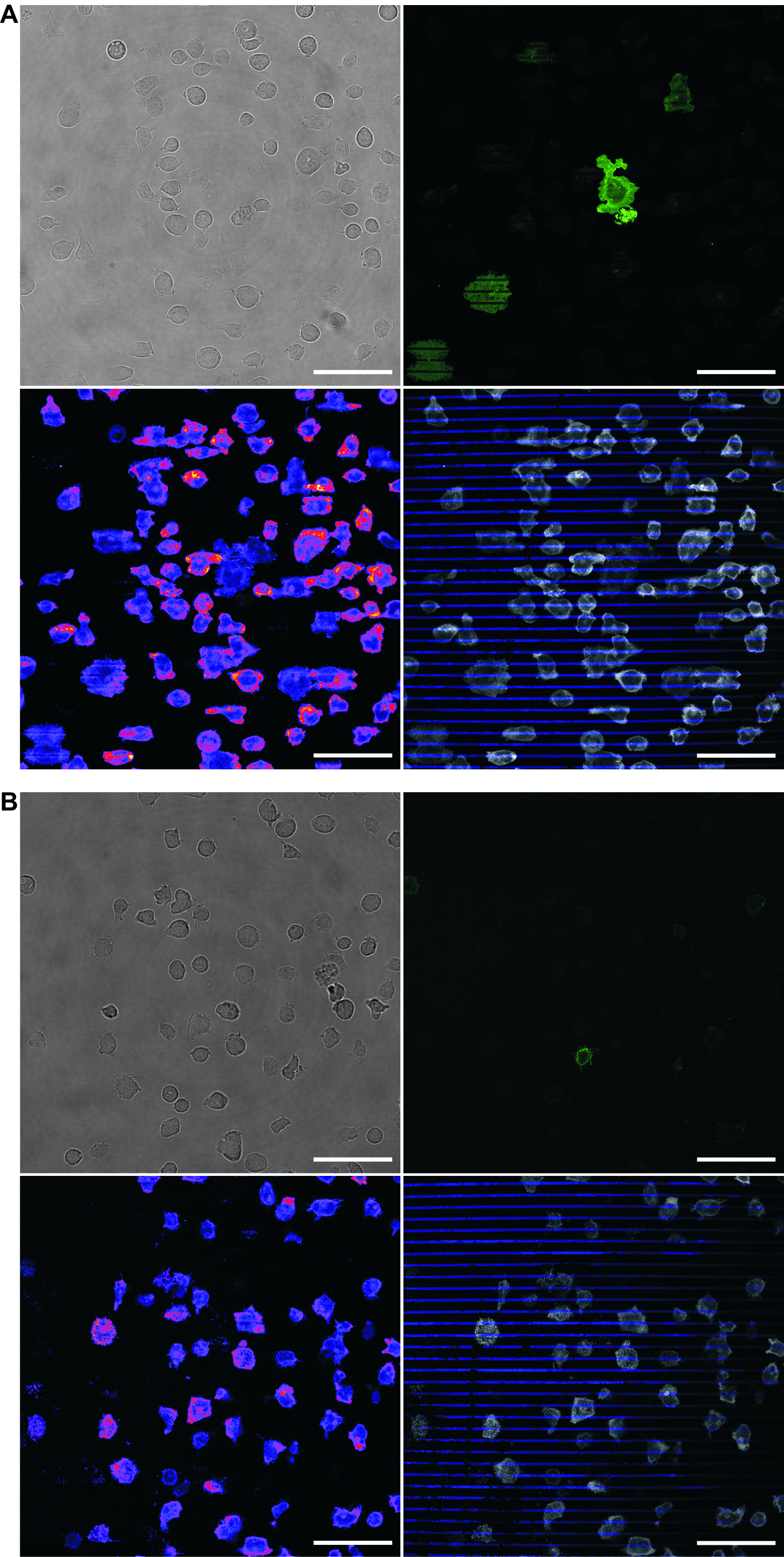

Supplement: Figure S4 — Tyrosine phosphorylation on control surfaces. CD28-GFP transfected Jurkat ACC-282 T cells were serum starved for 6 h and then incubated on striped surfaces for 10 minutes, fixed with 3% PFA and immunolabeled with αphosphotyrosine. Surfaces were functionalized using stamps coated with 25 µg/ml αCD3 (A) or unspecific IgG2a only (B). The remainder was subsequently overlaid with either 5 µg/ml αCD28 (A) or unspecific IgG2a only (B). Top left panels: transmission image; top right panels: CD28-GFP; bottom left: αphosphotyrosine; bottom right panels: overlay of the stamped pattern (blue) and the αphosphotyrosine label (grayscale). For a better comparison no adjustments were made to the contrast or brightness of the images. Scale bars 50 µm. (TIF) [file pone.0079277.s004.tif]

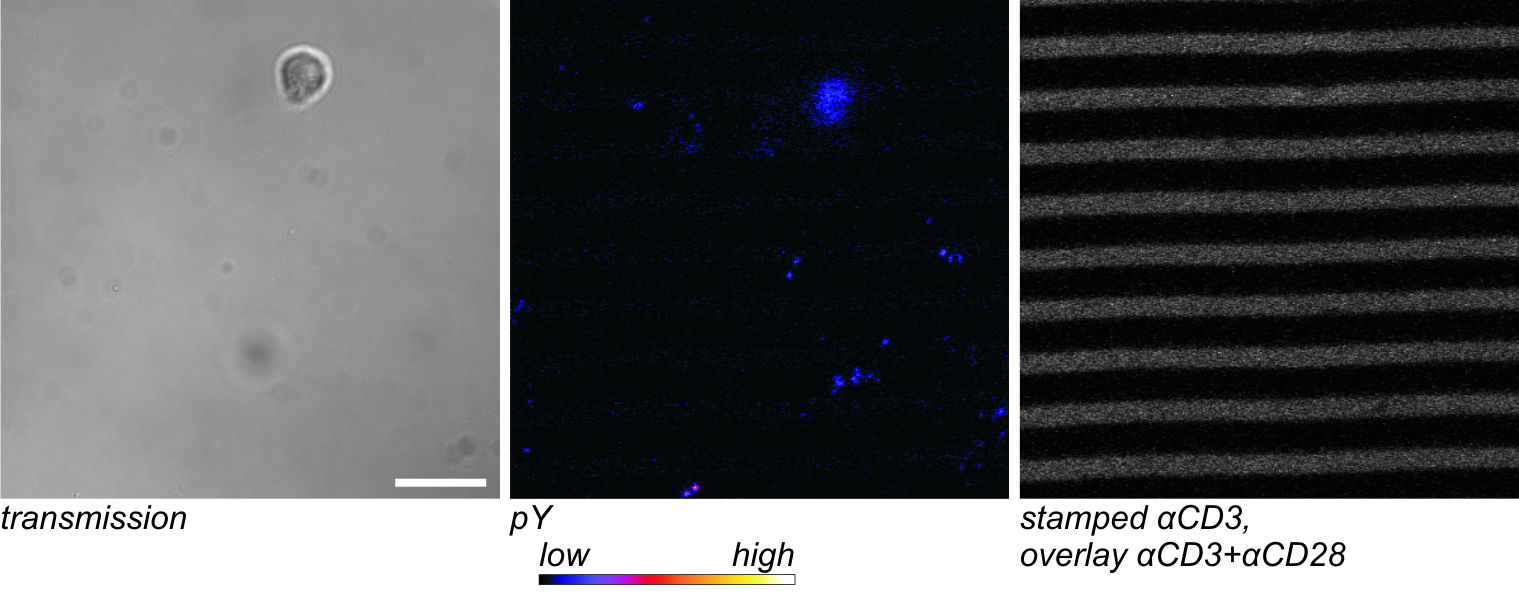

Supplement: Figure S5 — Reduced adherence and spreading of cells treated with cytochalasine D. Jurkat T cells were serum starved overnight and were treated with 10 µM cytochalasine D (Tocris Bioscience, Bristol, UK) 10 minutes prior to, and during incubation on striped surfaces. Surfaces were functionalized using stamps coated with 25 µg/ml αCD3 and overlaid with 2.5 µg/ml αCD3 + 2.5 µg/ml αCD28. Samples were immunolabeled with αphosphotyrosine. Images were acquired with a Zeiss LSM510 meta confocal laser scanning microscope using a 63×1.4 N.A. PLAN APO objective and 543 nm and 633 nm HeNe lasers (Carl Zeiss, Sliedrecht, The Netherlands). Panels from left to right: transmission image, immunolabel and stamped patterns. Scale bars 20 µm. (TIF) [file pone.0079277.s005.tif]

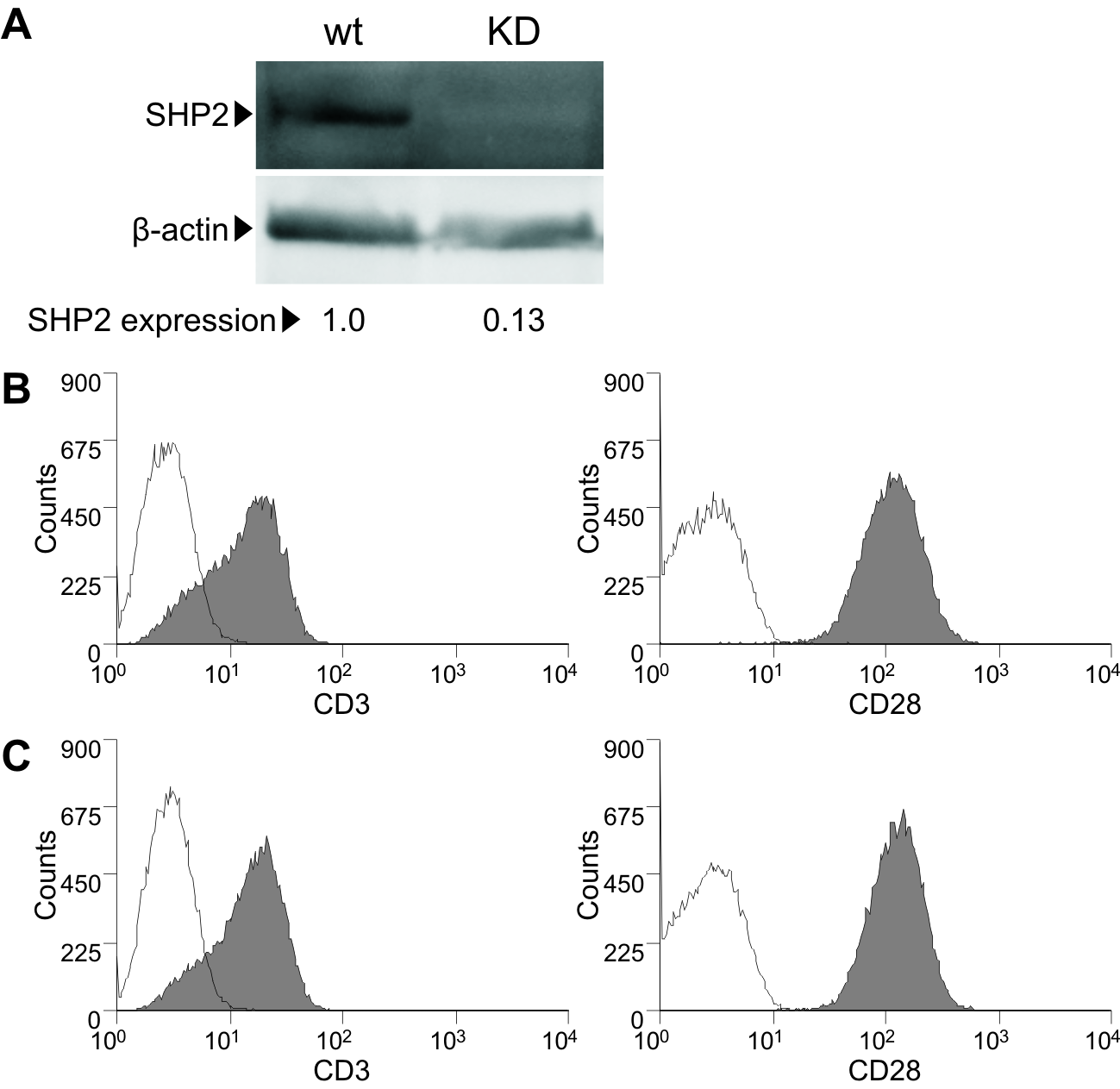

Supplement: Figure S6 — SHP2 expression in SHP2 knock-down cells is reduced to 13% of wild type levels but both lines express receptors at comparable levels. A) Total cell lysates of Jurkat E6.1 SHP2 KD cells and Jurkat E6.1 ‘wt’ cells were subjected to SDS-PAGE followed by immunoblotting of SHP2 expression using a SHP2 antibody (rabbit polyclonal, N-10) from Santa Cruz Biotechnology (Heidelberg, Germany) or β-actin antibodies (mouse monoclonal, AC-15, Sigma-Aldrich, Deisenhofen, Germany). After subsequent incubation with horseradish peroxidase–conjugated secondary antibodies, the blots were developed using Western Lightning chemiluminescence detection (Perkin Elmer Life Sciences, Boston, MA, USA) and quantitatively evaluated using a CCD camera-based system (LAS3000; Fujifilm, Düsseldorf, Germany). SHP2 levels were quantified in relation to β-actin levels. Below, SHP2 expression levels are given relative to levels in wt cells. B & C) Expression levels of CD3 (left panels, Zenon Alexa 488) and CD28 (right panels, Zenon Alexa 647) were determined with flow cytometry for SHP2 KD cells (A) and wt cells (B). The unfilled histograms show isotype controls while the filled histograms αCD3 and αCD28 labeled populations, respectively. (TIF) [file pone.0079277.s006.tif]

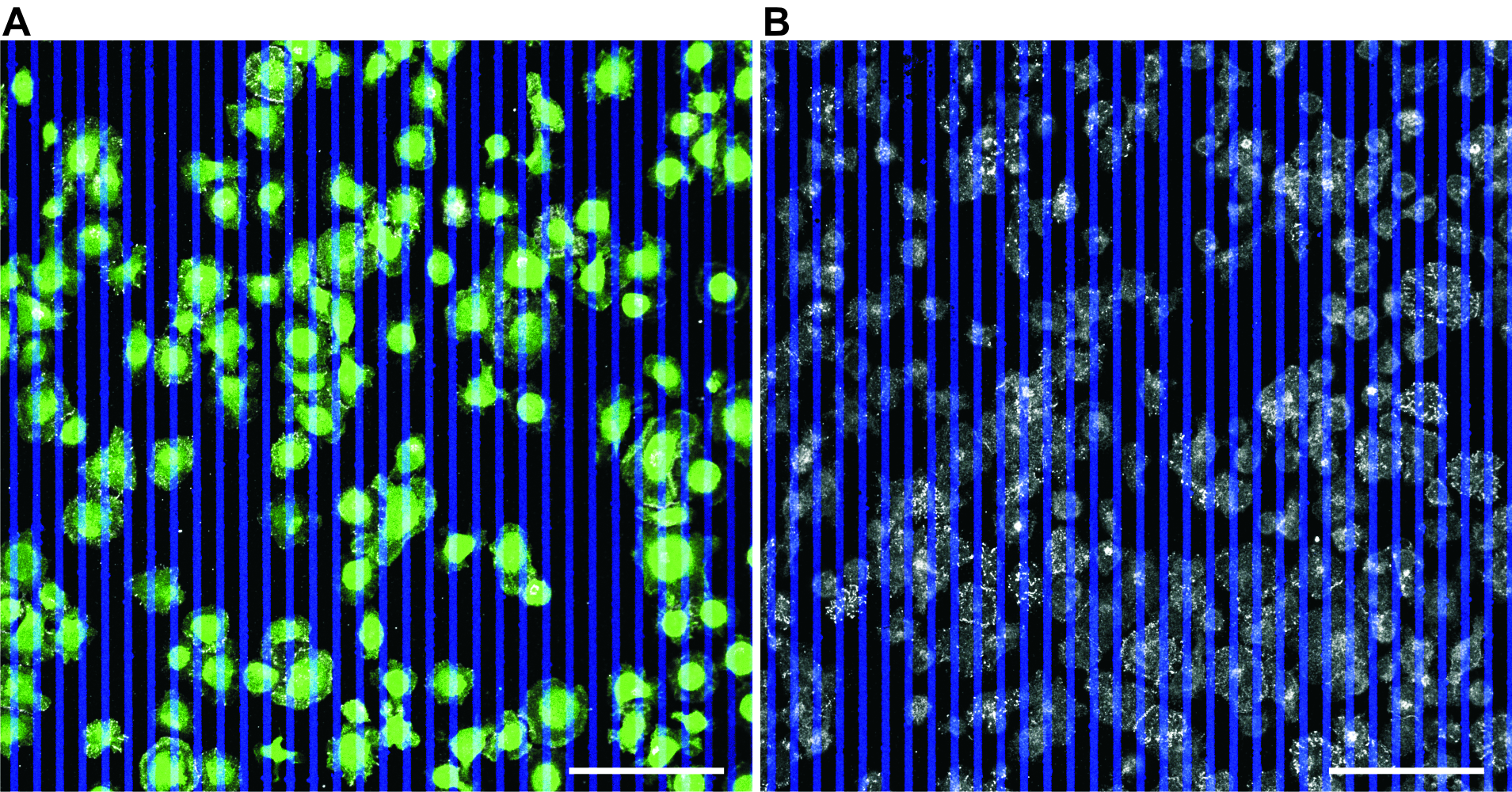

Supplement: Figure S7 — CFSE fluorescence (green) is retained by all cells after fixation, permeabilization and immunolabeling. Stamps coated with 25 µg/ml αCD3 were used to generate striped patterns (blue) which were overlaid with 2.5 µg/ml αCD3 + 2.5 µg/ml αCD28. Jurkat E6.1 ‘wild type’ cells were labeled with CFDA-SE (A) or mock labeled (B), serum starved over night and subsequently incubated on the micropatterned surfaces for 10 minutes, fixed with 3% PFA and immunolabeled with αphospho-PLCγ1 (grayscale). A & B were recorded with identical microscopy settings and all three channels are overlaid for both. For clarity, contrast and brightness were adjusted proportionally. Scale bar 50 µm. (TIF) [file pone.0079277.s007.tif]

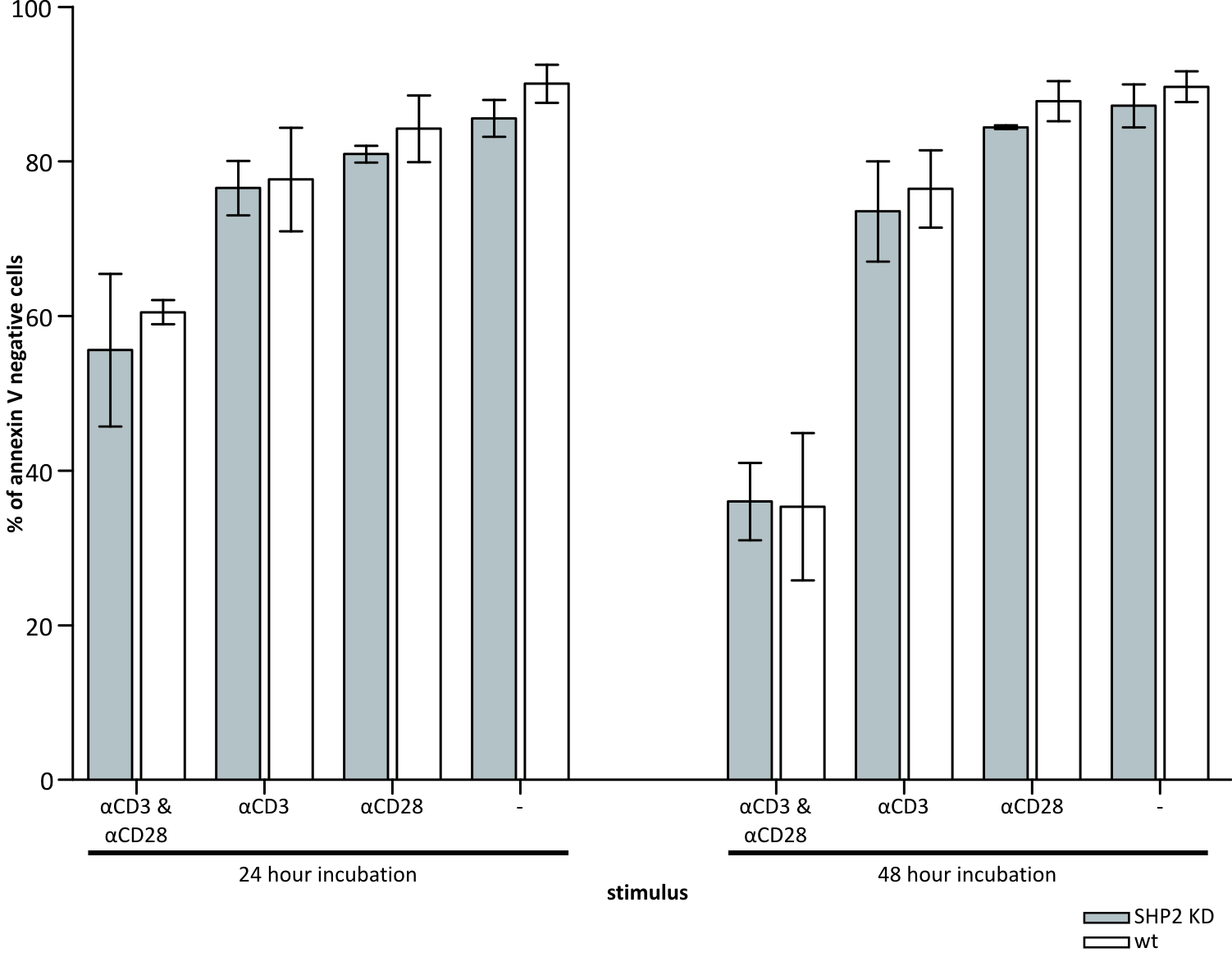

Supplement: Figure S8 — SHP2 knock down effect on phosphatidylserine exposure. Wells of a 96-well flat bottom plate were coated as described for the ELISA in the Materials and Methods section. In these wells 1•105 SHP2 KD or wt Jurkat T cells were stimulated with αCD3 & αCD28 (clone CD28.2; eBioscience, Frankfurt, Germany), αCD3 alone, αCD28 alone or were left unstimulated (-) for 24 (left) or 48 hours (right) at 37°C, 5% CO2 and under humidified conditions. Cells were subsequently stained with the Annexin V-PE 7-AAD Apoptosis Detection Kit I (BD Pharmingen, Heidelberg, Germany) using the suppliers protocol. Phosphatidylserine exposure was determined using a FACS Canto flow cytometer (BD Biosciences, Heidelberg, Germany) and characterizing 1•104 cells per sample. The graph shows the percentage of annexin V negative cells ± SEM of three independent experiments. (TIF) [file pone.0079277.s008.tif]
